# Supplementary material for: Are carbon emissions trading and green financial instruments synergistic? -Comprehensive quantitative research based on content analysis
Source: PLoS One. 2024 Mar 7;19(3):e0298601. doi: 10.1371/journal.pone.0298601 (PMC10919601; doi:10.1371/journal.pone.0298601)
Supplement: S1 Data — (ZIP) [file pone.0298601.s001.zip › Data/地级市Z_F工作报告.zip!/║╙─╧/╞╜╢Ñ╔╜╩╨/└┤╫╘╣1⁄2╓┌║┼í╛┬φ┐╦╩2╛▌═°í┐_2019.docx]

各位代表：

现在，我代表市人民政府，向大会报告工作，请予审议，并请各位政协委员和其他列席人士提出意见。

一、2018年工作回顾

过去的一年，面对严峻复杂的国际国内环境和艰巨繁重的改革发展稳定任务，在市委坚强领导下，全市上下以习近平新时代中国特色社会主义思想为指导，全面落实党的十九大精神和习近平总书记视察指导河南时的重要讲话精神，坚持稳中求进工作总基调，以新发展理念为引领，以高质量发展为根本方向，以供给侧结构性改革为主线，深入开展三大攻坚战，统筹推进“四个着力”，持续打好“四张牌”，着力推动“五大转型”，全市经济社会发展呈现总体平稳、稳中有进的良好态势。初步核算，全年生产总值增长7.5%，规模以上工业增加值增长7.8%，固定资产投资增长11.1%，社会消费品零售总额增长10.5%，一般公共预算收入增长12.2%、税比73.6%，城镇和农村居民人均可支配收入分别增长8.3%、8.8%。一年来，我们主要做了以下工作：

（一）千方百计抓关键稳运行，经济增长取得新成效。建立主要指标“红黄绿”预警机制，综合施策、科学调控，推动经济平稳增长。深化企业服务。坚持重点企业联席会议制度，及时帮助解决遇到的困难，全年累计为大企业解决重大问题89个。为176家科技型中小企业派驻了政府联络员，外来投资企业手续办理实现了全程代办制。扩大有效投资。健全推进机制，狠抓项目建设，争取上级项目206个、资金36.2亿元，重点项目完成投资850亿元、占全年计划的121.4%。强化要素保障。充实六大投资公司资本，总资产比改革前扩大3倍。平发集团获AA+信用评级，我市成为全省第4个拥有AA+投资公司的城市。银行业金融机构贷款新增142.2亿元，资本市场直接融资168.5亿元，1家企业在新三板挂牌，200家企业在中原股权交易中心挂牌。供应土地1.06万亩，重点项目建设用地实现应保尽保。优化发展环境。认真落实降低企业成本58条措施，减免企业税费34.2亿元；制定出台优化营商环境三年行动方案、促进非公有制经济健康发展的若干意见等政策，为实体经济发展营造良好环境。

（二）坚定不移调结构转方式，转型发展迈出新步伐。瞄准高质量发展主攻方向，深化供给侧结构性改革，加快构建现代产业体系。“一去三改”成效显著。压减30万吨煤炭产能，关停两座低效炼铁高炉，实施“三大改造”项目243个、完成投资146亿元，超额完成省定目标任务。中煤电气被评为国家级服务型制造云平台，6家企业被评为省级制造业与互联网融合发展试点示范项目，3家企业被评为省级智能工厂，2家企业被评为省绿色工厂。工业转型取得突破。中国尼龙城上升为省级战略，尼龙棉纺印染基地纳入全省布局，“1+7”制造业加快发展，尼龙新材料产业基地总体规划编制完成，总投资200亿元年产60万吨己内酰胺—聚合—纺丝一体化项目签约落地，年产7万吨尼龙6切片、4万吨尼龙66切片等项目建成投产，碳新材料、健康食品、电气装备、视光产品等新兴产业园建设步伐加快，产业集聚区固定资产投资增速居全省第2位、规模以上工业增加值增速居第7位。现代农业发展势头强劲。国家农业“两区”建设扎实推进，完成248万亩粮食生产功能区和重要农产品生产保护区划定，初步形成100万头生猪、10万头肉牛、5万头优质奶牛、1000万只绿色禽类等养殖产业集群；发展优质生态小麦45万亩、花生40.7万亩、大豆35万亩，形成近2万吨大豆精深加工能力；与正大集团、中民投集团合作的农业项目加快推进；烟草业转型升级综合指标实现全省“四连冠”。现代服务业加快发展。新增国家A级旅游景区8家，现代物流园、舒山健康养老园区被确定为省级服务业专业园区，电子商务交易额317.9亿元、增长46.8%，网络零售额89.8亿元、增长38.1%，跨境电商进出口总额10.5亿元、增长44.9%。

（三）全力以赴补短板强弱项，三大攻坚打出新局面。坚持把打好三大攻坚战作为全面建成小康社会硬任务，统筹安排、强力推动，实现良好开局。防范化解重大风险扎实有效。开展互联网金融风险专项整治，稳妥处置重点领域和企业风险隐患，农信社不良贷款逐步妥善化解，平顶山银行实现稳健运行。化解问题楼盘255个、占任务量的84%，其中中心城区化解率达95%。非法集资案件加快处置，政府债务控制在省定标准以内。精准脱贫深入推进。扎实推进十大工程，投入专项扶贫资金14.3亿元，扶贫小额信贷累计贷款27.4亿元，完成1.4万户危房改造、1万人易地搬迁安置、382个行政村饮水安全巩固提升工程，产业覆盖率达99.4%，4.7万人实现脱贫，叶县有望脱贫摘帽。污染防治强力推进。大力开展大气、水、土壤污染防治，突出抓好中央和省环保督察反馈意见整改落实，35家企业完成超低排放改造，20家企业创建为“绿色企业”，PM10、PM2.5平均浓度和优良天数改善幅度分别居全省第7位、第9位、第8位，城市集中式饮用水水源地水质达标率100%、并列全省第1位，获省水环境质量生态补偿金数额居全省第2位，土壤环境质量总体稳定。

（四）坚持不懈促改革扩开放，经济发展注入新动能。深入推进国有企业改革，提前1个月完成“僵尸企业”处置任务，被评为全省国企改革攻坚工作先进市。持续深化行政服务改革，机构改革平稳有序推进。加快实施“一次办妥”改革，集中解决政务服务事项“联、通、办”问题，市、县、乡三级政务外网实现全覆盖，不动产登记全面实现网上预约、申请和查询。纵深推进“多证合一”改革，新增市场主体5.9万户、增长26.7%。农村土地承包经营权确权颁证工作全面完成，集体产权制度改革稳步推进。平顶山—宁波港海铁联运“沿江班列”每周一班，打通了连接海上丝绸之路的通道。全面实施签约项目全程代办服务制，举办中国尼龙新材料产业技术发展大会等活动，实际吸收外资增长4.1%、居全省第5位，实际到位省外资金579.3亿元、居第10位。深入实施创新驱动发展战略，加大“四个一批”建设力度，新增科技型中小企业170家、高新技术企业24家，新增省级工程技术研究中心、院士工作站、新型研发机构、农业科技园区等高层次创新平台25家。高新区获批国家创新创业特色载体，12家企业入选郑洛新国家自主创新示范区首批辐射点。

（五）持之以恒抓创建强引领，城乡面貌得到新改善。强力推进“四城联创”，顺利通过国家森林城市复查验收、国家卫生城市复审暗访和省级节水型城市创建考核验收，文明城市创建扎实推进。颁布实施我市第一部地方性法规——《平顶山市城乡规划建设管理条例》，编制完成全域规划初步方案和26项城市专项规划。深入实施百城建设提质工程，开工断头路60条、建成18条，新增停车位5000余个，优化改造道路交叉口39个，新建改建城乡公厕455座，完成15个游园提档升级、18座立交桥立体绿化，湛河主河道综合治理全面完成。新增供热面积300万平方米，集中供热普及率达70.7%；新建燃气管网57公里，中心城区天然气普及率达97%。取缔中心城区6个马路市场和27个大型货车停车场，拆违拆迁936万平方米，完成245个老旧社区基础改造、25个老旧社区全面改造。争取棚户区改造安置规模6.7万套，居全省第4位。大西环、平宝快速通道“公跨铁”等项目加快推进，农村公路完成建设里程586.7公里，郏县、叶县获全省“四好农村公路”示范县称号。全面落实河长制湖长制，严厉整治非法采砂，清理河道垃圾8.9万立方米，疏浚河道242公里。加快推进国土绿化提速行动，完成生态林建设43.2万亩，全市绿化覆盖率达40.8%。开展“千村整治、百村示范”，推进全域农村垃圾综合治理“户分类、村收集、乡转运、县处理”，完成农村户用厕所无害化改造10.5万户，硬化村庄道路2500公里，村容村貌明显提升。

（六）倾心尽力惠民生保稳定，百姓福祉实现新提升。财政民生支出增长12.4%，占一般公共预算支出的76.5%，重点民生实事落地见效。着力做好重点群体人员就业和农民工返乡下乡创业，城镇新增就业7.9万人，返乡下乡创业8.7万人。城乡居民基本养老保险参保率达97.9%，16.9万建档立卡贫困人口全部纳入城乡居民养老保险覆盖范围。新建改扩建幼儿园15所，学前三年毛入园率达91%；义务教育阶段学校大班额和超大班额比例分别下降19.1个、9.5个百分点，“全面改薄”工作居全省第1位；市实验高中、市46中搬至新校区；河南城建学院部分专业实现一本招生，平顶山学院在省示范性应用技术类型本科院校考评中位居前列。稳步推进公立医院改革，开展现代医院管理制度与薪酬制度改革试点，“三医”联动改革和分级诊疗建设取得新突破，“互联网+医疗健康”工作走在全省前列。基层公共文化服务体系加快建设，文化遗产保护力度持续加大，全民健身蓬勃开展。平顶山籍小型客车宁洛高速城区段实行免费通行。严格落实安全生产责任制，深化重点行业领域安全专项治理，安全生产形势持续稳定。推进食品安全市建设，食品药品安全监管继续加强。深入开展扫黑除恶专项斗争，依法打击各类违法犯罪活动，认真做好信访工作，公众安全感和执法满意度“双提升”，社会大局保持和谐稳定。

一年来，我们深入开展干部作风大整顿，扎实做好中央巡视反馈意见整改落实，强化审计监督，持续纠治“四风”，全面从严治党取得新成效。自觉接受人大监督、政协民主监督和社会监督，认真办理人大代表建议和政协委员提案，法治政府建设取得新进展。加强国防教育和国防后备力量及人民防空建设，支持军队改革发展，推进军民融合深度发展，驻平解放军、武警部队和民兵预备役人员为地方发展作出新贡献。民族宗教工作创新推进。妇女儿童、残疾人、红十字、慈善等事业健康发展。外事、侨务、对台、统计、史志、档案、气象、地震等工作取得新成绩。

各位代表！

回顾过去的一年，我们更加深切体会到，做好各项工作，必须坚持党对一切工作的领导，树牢“四个意识”，坚定“四个自信”，践行“两个维护”，自觉坚持党中央集中统一领导，确保党中央各项决策部署落到实处、见到实效；必须坚持发展第一要务，深入贯彻落实新发展理念，坚定不移走高质量转型发展之路，保持经济社会持续健康发展；必须坚持底线思维，增强忧患意识，提高防控能力，着力防范化解重大风险，为经济社会发展奠定坚实基础；必须坚持深化改革开放，以改革破难题，以开放求突破，以创新促发展，充分激发市场活力和社会创造力；必须坚持务实重干、拼搏进取，撸起袖子加油干，扑下身子抓落实，全力推动各项工作晋位次争上游走前列；必须坚持以人民为中心，践行党的根本宗旨，持续办好民生实事，让改革发展成果更多更公平惠及全市人民。

各位代表！

成绩来之不易，经验弥足珍贵。这些成绩的取得，最重要、最根本的是有以习近平同志为核心的党中央正确领导，有习近平新时代中国特色社会主义思想的科学指导，也是全市人民在省委、省政府和市委领导下和衷共济、攻坚克难的结果。在此，我代表市人民政府，向全市广大干部群众，向驻平部队和武警官兵，向各民主党派、人民团体和社会各界人士致以崇高敬意！向关心支持平顶山发展的海内外友好人士表示诚挚感谢！

在看到成绩的同时，我们也清醒认识到，经济社会发展中还面临不少困难和问题。一是经济下行压力明显增大，市场需求增长放缓，企业生产要素成本较高，营商环境与经济发达地区相比仍有不小差距，实体经济发展面临较多困难。二是转型发展任务艰巨，创新能力不够强，新增长点不够多，县域经济、民营经济和服务业发展不充分，发展新动能不足。三是城市治理比较粗放，规划管控体系还不完善，城市功能还有许多欠账，环境污染、交通拥堵、停车难等问题尚未有效解决，就业、教育、医疗、养老等民生领域仍有不少短板。四是风险隐患依然较多，非法集资、政府债务、安全生产等领域风险隐患不容忽视。五是政府工作仍有不足，有些改革和政策措施落实不到位，有些干部观念落后、能力不够，市场意识、创新精神、开放思维不强，不想为、不敢为、不善为依然存在，个别领域不正之风和腐败现象仍时有发生。对此，我们一定高度重视、切实解决，努力把各项工作做得更好。

二、2019年工作安排

今年是新中国成立70周年，是全面建成小康社会、实现第一个百年奋斗目标的关键之年，做好今年的工作意义重大。当前，我国发展仍处于重要战略机遇期，我市推动高质量发展尽管面临不少压力和挑战，但也具备诸多有利条件，我们既有国家政策的支持，又有自身优越的资源禀赋、坚实的产业基础。特别是，我市在被确定为国家农业“两区”的基础上，去年成功纳入淮河生态经济带，尼龙新材料产业成为省重点培育发展产业，今年有望成为第二批国家老工业基地和资源型城市产业转型升级示范区试点，将为我市带来政策、资金、项目等诸多利好；干部作风大整顿和电视问政的深入开展，更是激发了全市干部新时代新担当新作为的强大正能量。只要我们坚定信心、鼓足干劲，紧盯目标、锲而不舍，就一定能够拼出高质量发展新天地、开创跨越赶超新局面。

今年政府工作要认真落实市委九届八次全会要求：以习近平新时代中国特色社会主义思想为指导，全面贯彻党的十九大、十九届二中、三中全会和习近平总书记视察指导河南时重要讲话精神，认真落实中央经济工作会议和省委十届八次全会决策部署，统筹推进“五位一体”总体布局，协调推进“四个全面”战略布局，坚持稳中求进工作总基调，坚持新发展理念，坚持推动高质量发展，坚持以供给侧结构性改革为主线，坚持深化市场化改革、扩大高水平开放，贯彻巩固、增强、提升、畅通八字方针，继续打好三大攻坚战，持续推进“四个着力”、打好“四张牌”，全面加快“五大转型”，激发微观主体活力，统筹推进稳增长、促改革、调结构、惠民生、防风险、保稳定工作，进一步落实“六稳”要求，提振市场信心，增强人民群众获得感、幸福感、安全感，保持经济平稳健康发展和社会大局稳定，持续提升“八个度”，在综合实力高质量重返全省第一方阵征程中迈出更大步伐，以优异成绩庆祝中华人民共和国成立70周年。

今年经济社会发展主要预期目标是：生产总值增长7.5%以上，全员劳动生产率增长7%，一般公共预算收入增长8%、税比达到73%以上，规模以上工业增加值增长7.5%以上，固定资产投资增长8.5%，社会消费品零售总额增长10%左右，居民消费价格涨幅控制在3%左右，经济结构进一步优化，质量效益明显提升。创新发展取得新成果，研究与试验发展经费投入强度达到1.5%，高技术产业、战略性新兴产业占比进一步提升。协调发展取得新成效，常住人口城镇化率提高1.5个百分点以上，人均预期寿命持续提高，九年义务教育巩固率达到95%，医疗卫生、文化、体育等社会事业全面进步。绿色发展取得新提升，单位生产总值能耗下降3%以上，主要污染物排放量完成省下达目标。开放发展取得新突破，进出口保持平稳增长，海关技术中心加快建设，营商环境进一步优化。共享发展取得新进步，居民人均可支配收入增长8%，4.12万农村贫困人口实现脱贫，城镇新增就业6万人以上，城镇调查失业率和城镇登记失业率分别控制在5.5%以内和4%以内。以上目标，更加凸显了新发展理念和高质量发展要求，有利于把工作重点放在推进高质量发展上来。

落实总体要求，实现预期目标，必须把握好“形”与“势”、“危”与“机”、“供”与“需”、“稳”与“进”四个关系，坚决把发展扭住、把重点抓住、把风险管住、把民生兜住，倡树“两学两比两争”，持续晋位次争上游走前列，推动各项工作落地见效。

今年重点抓好八个方面的工作：

（一）激发市场需求潜力，推动经济稳定增长。努力扩大市场需求，支持实体经济发展，保持经济健康运行。

激活区域市场需求。一方面，抓住国家促进形成强大国内市场的机遇，努力拓展消费需求。加快推动消费升级，满足人民群众中高端服务消费和物质产品消费需求。整合提升文化旅游资源，完善旅游设施，提升服务品质，打造精品路线，推进旅游业转型发展。梳理民间资本进入养老、医疗、家政、托幼等领域的堵点和难点，放宽消费领域市场准入，推动医养融合化发展、居家社区养老服务社会化发展。落实个人所得税专项附加扣除政策和最低工资保障制度，拓宽居民劳动收入和财产性收入渠道，增强消费能力。持续开展质量提升行动，严厉打击侵害消费者权益行为，不断改善消费环境，让老百姓吃得放心、穿得称心、用得舒心。另一方面，围绕产业转型升级、基础设施建设和民生改善等重点领域，持续扩大有效投资。紧盯国家基础设施补短板指导意见确定的九大重点领域，加强项目谋划和储备，积极争取政策资金支持。着力抓好414个重点项目建设，完成投资890亿元以上。深入实施促进民间投资健康发展专项行动，放宽民间资本进入资源开发、交通、市政等领域限制，充分激发民间投资活力。

稳定工业经济增长。开展企业服务年活动，坚持重点企业联席会议制度，持续实施企业成长促进行动，深入开展产销、银企、用工和产学研对接，切实为企业排忧解难。支持平煤神马集团、平高集团、舞钢公司等大型企业发展，推动神鹰化工、翔隆不锈钢、铁福来、国玺超纯、泰田重工等中小型科技企业做大做强，加快60万吨己内酰胺—聚合—纺丝一体化、40万吨聚碳酸酯、20万吨己内酰胺、15万吨环己酮、8万吨尼龙6印染一期、中石油叶县盐腔储气库、碳新材料产业园、煤焦化工产业园等项目建设，推动获国家专利金奖的抗艾滋病新药阿兹夫定实现产业化，为工业发展注入强劲动力。推进制造业与现代服务业深度融合，大力发展工业互联网，加快高新区跨境装备制造产业园区建设，深化大数据在制造业中的应用。

促进非公经济发展。认真贯彻习近平总书记民营企业座谈会重要讲话精神，深入落实促进非公有制经济发展“3+N”政策，打破各种“弹簧门”“玻璃门”“旋转门”，消除民营企业在准入许可、工程投标、银行融资等方面的不公平待遇，提振民营企业投资和发展信心。构建亲清新型政商关系，营造法制化制度环境，畅通企业家投诉渠道，依法保护其合法权益。开展民营企业和小微企业“百千万”行动，出台金融助推中小企业成长计划，有效破解民营企业和小微企业融资难融资贵问题。认真开展专项清欠行动，切实解决政府部门和国有企业拖欠民营企业账款问题。培育壮大民营企业家队伍，大力弘扬企业家精神。引导提升企业素质，推进民营企业制度创新、科技创新、产品创新、模式创新，促进二次创业和转型发展。

加快县域经济发展。全面贯彻习近平总书记关于县域治理“三起来”的重大要求，发挥县域优势，激发县域活力，增强县域综合实力。实施产业集聚区高质量发展专项行动，加快解决集聚效应不明显、主导产业不突出、体制机制不活等问题，推进产业集聚区二次创业。开展服务业“两区”和专业园区提质升级专项行动，再培育建设一批现代服务业专业园区。加大金融、创业培训、社会保障等政策支持力度，吸引返乡人才和乡贤名流就业创业，为县域经济发展注入新动力。把提高户籍人口城镇化率作为促进县域经济发展的重要举措，促进有能力在城镇稳定就业和生活的常住人口有序实现市民化。

（二）贯彻落实“八字方针”，促进经济提质增效。按照“八字方针”总要求，深化供给侧结构性改革，不断提升经济发展质量水平。

巩固“去降补”成果。严格执行环保、能耗、质量、安全、技术等标准，综合运用市场化、法治化手段退出低效过剩产能，拆除两座低效炼铁高炉及相关设备，淘汰改造技术落后、布局不合理煤电机组。持续开展“退城进园”，加快推进城镇人口密集区重污染企业搬迁改造。支持老城区和产业集聚区盘活企业闲置土地和厂房，发展高科技、高附加值产业。抓好“僵尸企业”处置后续工作，做好债务化解、破产审理、税收支持、资产处置等工作。加大对“破、立、降”奖补支持力度，落实好去产能职工安置、矿权土地、债务处置等政策，形成正向激励和优胜劣汰的政策导向。

增强微观主体活力。建立公平开放透明的市场规则和法治化营商环境，破除各类要素流动壁垒，发挥企业和企业家主观能动性，发展更多优质企业。加快诚信体系建设，完善守信褒奖、失信联合惩戒机制，坚决纠正一些地方和部门“新官不理旧账”违约做法。深化城镇规划区内建设用地、农村耕地、农村集体建设用地改革，加快土地等要素市场化步伐。大力实施企业上市“121”行动计划，支持更多企业通过多层次资本市场进行融资。全面落实国家和省更大规模减税降费政策，进一步清理规范涉企收费，加大力度降低企业社保费率，加快推进电力直接交易，切实减轻企业负担，让企业有实打实的获得感。

提升产业链水平。以产业高端化、技术尖端化、质量顶端化为目标，更加注重建链、补链、延链、强链，加快构建现代产业体系。突出中国尼龙城建设。认真落实省尼龙新材料产业三年行动计划，创新尼龙新材料产业集聚区发展体制，优化“一区三园”功能布局，加快基础设施建设，强化要素保障，大力发展下游尼龙织造、工程塑料产业集群，打造世界一流的尼龙新材料产业基地。推动七大支柱产业发展。依托平高集团、平煤神马集团大力发展储能产业，以高压电气、矿山机械、摩托机车、环保设备等为重点建设电气装备城，统筹抓好盐化工及PC、特钢不锈钢、煤焦油深加工、碳新材料、健康食品等产业发展，推动制造业高质量发展。抓好“三大改造”。以创建国家老工业基地和资源型城市产业转型升级示范区为抓手，深入实施12个重点产业转型发展专项方案，加快推进绿色化改造扩面提标、智能化改造赶超跨越、技术改造提升水平，实施“三大改造”项目120个，完成投资150亿元以上。深入推进质量强市建设，强化标准引领作用，弘扬劳模精神和工匠精神，支持企业增品种、提品质、创品牌。

畅通经济循环系统。着力畅通生产、流通、分配、消费循环，加快建设统一开放、竞争有序的现代市场体系。深入推进普惠金融建设，加快金融产品与服务创新，积极引进银行、保险、证券等金融机构，鼓励域外金融机构在我市设立分支机构，不断丰富金融供给，提高金融服务实体经济能力。出台促进物流业转型发展具体支持政策，加强与中民投集团的合作，加快推进现代物流产业园、泰顺达电商仓储物流园、多式联运智慧物流港、华耀城等项目建设，大力发展冷链物流、快递物流、电商物流，构建覆盖城乡的分拨配送物流网络。加强与阿里国际站等国内外知名电商平台合作，培育本土电商平台，推进跨境电子商务产业园区建设。大力推进电子商务进农村，打通农产品网上销售通道。

（三）继续打好三大攻坚战，着力夯实发展基础。聚焦突出问题，打好重点战役，全力攻坚，务求实效。

打好防范化解重大风险攻坚战。紧盯重点风险领域，着力解决突出问题，坚决守住不发生区域性系统性风险底线。防控地方债务风险。加强政府债务审计，规范政府举债融资机制，健全风险评估和防控长效机制，加大违规举债问责力度，坚决杜绝各类违法违规举债行为。防范化解金融风险。全面加强地方金融监管，做好风险点防控，扎实开展互联网金融风险专项整治，支持银行业金融机构处置不良资产，支持平顶山银行稳固发展。保持对非法集资高压态势，加大陈案攻坚力度，坚决遏制新发案件。有序推进问题楼盘化解，有效防范房地产领域金融风险。稳妥化解企业风险。深入推进市场化“债转股”，引导企业强化风险意识、优化债务结构，加快资产证券化步伐。完善重点风险企业台账，抓好企业间担保链风险防范，防止风险传导蔓延。有效防范社会风险。严格落实安全生产责任制，加快推进风险隐患双重预防体系建设，强化源头管控治理，坚决防范遏制各类事故发生。增强防洪抗灾能力，防范自然灾害风险。健全应急管理体系，做好突发公共事件预防处置。

打好精准脱贫攻坚战。坚持精准方略，把提高脱贫质量放在首位，严格落实市县主体责任、部门协调责任、乡村包干责任、驻村帮扶责任，确保4.12万人脱贫、120个贫困村退出、鲁山县脱贫摘帽。落实重点任务。按照脱贫攻坚提质增效32条工作标准，深入推进脱贫攻坚四场硬仗、六大行动、四项工程，打好脱贫攻坚总攻战。严格落实各项扶贫政策，把产业扶贫放在根本性和战略性位置，进一步完善工作机制、强化激励考核，优化带贫模式、搞好分类施策，不断提高产业带贫成效；加快实施危房改造清零行动，敦促有房子女将老人接入安全住房共同生活，做到危房不住人、住人无危房。开展重点攻坚。聚焦脱贫摘帽县、深度贫困村、特殊贫困群体，加大政策、资金、项目、人才、科技等资源要素支持力度，深入开展百企帮百村、万商扶万家等活动，做好结对帮扶、定点帮扶、校地帮扶等工作，重点解决好实现“两不愁三保障”面临的突出问题。加强脱贫攻坚和乡村振兴的统筹衔接，统筹贫困地区和非贫困地区发展，着力解决收入水平略高于建档立卡贫困户的群体发展致富问题，做好贫困“边缘户”帮扶工作。巩固脱贫成果。加强政策宣传和教育引导，加大就业技能和实用技术培训力度，改进帮扶方式方法，建立脱贫正向激励和稳定脱贫长效机制，减少和防止贫困人口返贫。

打好污染防治攻坚战。扭住改善环境质量这个核心，严格落实党政领导责任、企业主体责任、部门监管责任，持续加大环境治理力度，扎实开展中央和省环保督察反馈问题整改，努力推动生态转型，着力建设绿色矿业城。坚决打赢蓝天保卫战。坚持精准施策、标本兼治，突出重点区域、重点行业、重点污染物的监管，持续狠抓“六控”，突出治理“两散”，优化调整“四个结构”，深入开展工业企业绿色改造，着力抓好柴油货车污染治理，大力发展城市公共交通，彻底整治北部矿区污染，解决好结构性、季节性、区域性污染问题，确保完成省定空气质量目标任务。全面打赢碧水保卫战。严格落实河长制湖长制，深入实施水污染防治行动计划，强化重点流域和河流治理，加强城市生态水系建设和饮用水源地保护，消除城区黑臭水体，确保集中式饮用水水源地水质达标率保持100%、出境断面水质综合达标率完成省定目标。扎实推进净土保卫战。深入开展全国第二次污染源普查，实施耕地土壤环境质量分类管理，加强重金属污染、农业面源污染防治和固体废物处理，推进受污染耕地安全利用和治理修复，严格保护未污染土壤。

（四）深化改革开放创新，增强高质量发展新动能。强化改革推动、开放带动、创新驱动，最大限度激发发展动力活力。

以全面深化改革激发动力。推进政府机构改革。深入推进市县机构改革，改革机构设置，优化职能配置，提高行政效能。深化“放管服”改革，加快建设网上政务服务平台和“互联网+监管”系统，加强“双随机、一公开”监管，把“一网通办”前提下的“最多跑一次”改革落到实处，提升政府数字化管理水平，最大限度利企便民。纵深推进“多证合一”，全面推开“证照分离”改革，加快推进企业登记注册全程电子化。打好国企改革“扫荡战”。在做好“僵尸企业”处置后续工作的同时，持续深化企业产权结构、组织结构、治理结构改革，深入推进混合所有制改革，全面完成市属企业公司制改制。持续完善国有资产监管，理顺金融资本管理体制机制，推进智能化国资监管平台建设，支持平发集团开展国有资本投资运营公司试点，加快实现由管企业向管资本转变。深化投融资体制改革。继续优化投资项目审批，大力推广多评合一、企业投资项目承诺制等模式，一般性企业投资项目全流程审批时限压减至100个工作日以内。完善市属投资公司经营模式，支持其参与土地一级市场开发、广告牌和停车位经营、重要资源特许经营，不断壮大公司实力。用好用足地方政府债券政策，支持发行企业债券、双创专项金融债券和孵化专项债券。发挥好地方政府融资担保体系作用，规范发展政府和社会资本合作项目。健全生态环保体制。完善月度生态补偿、绿色环保调度、污染物排放许可证等制度。实施自然资源资产产权制度和有偿使用制度改革。推进生态环境机构监测监察执法垂直管理体制改革，抓好生态环境保护综合行政执法改革。深化财税体制改革。加快构建现代预算制度，全面实施预算绩效管理，提高财政资金配置效率和使用效益。持续推进高新区管理体制改革，继续深化教育、医疗、警务管理、城市综合执法等改革。

以全方位扩大开放增添动力。抓好招商引资。坚持把开放招商作为一号工程，转变招商理念、创新招商方式，围绕主导产业和新技术、新业态，持续开展驻地招商、精准招商、集群招商，积极推行股权招商、并购招商、飞地招商等新模式，再引进一批战略性、龙头型项目。健全招商引资工作机制，完善督查考评办法，充分调动各级各部门招商引资积极性。发展对外贸易。落实好国家、省促进外贸发展的优惠政策，加大对企业的跟踪服务力度，持续推进出口基地建设。充分发挥平顶山—宁波港海铁联运班列作用，深度融入“一带一路”，积极开拓国际市场。加快海关技术中心等平台建设，为外贸企业提供高效便捷服务。优化营商环境。深入落实优化营商环境实施意见，启动营商环境评价；全面落实准入前国民待遇加负面清单管理制度，建立外商投资企业投诉工作机制，严格企业知识产权保护；进一步完善招商引资签约项目全程代办服务制，将代办服务延伸至项目建设全过程，营造良好营商环境。

以创新驱动培育发展新动力。完善科技创新体系。持续抓好“四个一批”建设，实施创新龙头企业提升引领、高新技术企业倍增、“科技小巨人”企业培育工程，培育高新技术企业20家、科技型中小企业50家，建设省级以上创新平台10家以上，争创5家省级双创载体。深入实施“鹰城英才计划”，落实人才政策待遇，重视解决住房、子女教育等实际问题，建设高层次创新人才团队。提升科技创新能力。推进国家创新型城市、国家创新驱动示范市、国家知识产权示范市创建，加大财政投入力度，引导企业加大研发投入，围绕产业链布局创新链，在尼龙化工、生物医药、新能源、装备制造等领域，集中实施一批具有重大支撑性、先导性的科技专项，在核心关键环节实现技术突破，带动产业创新发展。健全创新体制机制。加快科技创新服务平台建设，支持县（市、区）依托产业集聚区建设科技创新综合体，发展一批科技服务机构，提升科技创新服务能力。深化项目评审、人才评价、机构评估改革，加快科技成果评价改革和科技成果所有权改革，落实科技成果转化激励改革举措，建立试错、容错、纠错机制，让创新者敢于创新、安于创新、乐于创新。

（五）深入推进百城建设提质工程，着力提升城镇化质量。坚持以人的城镇化为核心，加快解决发展方式、空间、环境、要素等问题，不断提高城镇化发展质量。

优化城镇布局。加强城镇发展战略研究，科学编制国土空间规划和城市总体规划，继续推进老城区功能疏解和改造提升，加快完善城乡一体化示范区功能，加强高铁片区等九大片区开发建设，强化市区东部基础设施建设，推进高新区、湛南新城、昆北新城、尼龙城功能互补、产业融合，不断优化中心城区功能布局，提高辐射带动能力。推动宝丰、叶县、鲁山与中心城区一体化发展，增强汝州、舞钢、郏县支撑地区发展能力，规范引导特色小镇和小城镇布局建设。

提升城市综合承载能力。进一步完善停车场、游园、公厕、文体设施等功能配套，合理布局学校、医院、农贸市场、商业网点等公共服务设施，加快市中医院新城区分院、市第一人民医院新院区、四馆一中心、平煤神马集团研发中心、市委党校和市一高迁建等项目建设，不断提升城市功能品质。持续实施打通断头路行动计划，加快推进龙翔大道等城市道路改造延伸，不断提升通行能力。加快城市污水处理和中水利用工程建设，确保中心城区污水处理率达到89.5%以上。

持续开展城市创建。突出“污、堵、脏、差”四治，加快推进智慧城市建设，着力抓好市场提档改造、老旧小区改造、“双违”拆迁、路口优化改造、市花市树普及推广、城区生活垃圾分类等重点工作，深化城市清洁、交通文明畅通和市民思想道德素质提升行动，不断提升城市精细化管理水平，巩固提升城市创建成果，推进全国文明城市创建取得积极进展。

（六）扎实推进乡村振兴战略，推动“三农”工作再上新台阶。坚持农业农村优先发展，加快推进产业、人才、文化、生态、组织“五个振兴”，推动实现农业强、农村美、农民富。

大力发展现代农业。以建设绿色食品城为目标，以国家农业“两区”建设为抓手，建立健全农业绿色发展制度，持续实施“循环农业+品牌农业+协同农业”发展战略，突出叶县、郏县、鲁山等重点区域，引进培育一批龙头企业，对标国际国内先进标准，增加绿色、优质、高端农产品供给，巩固上海目标市场，拓展欧洲、日本等发达国家市场，打响具有平顶山特色的绿色农业品牌。认真落实粮食安全行政首长责任制，加强高标准粮田建设和中低产田改造，抓好农田水利、农机、气象等基础设施建设，夯实粮食稳产基础。积极调整“粮经饲”结构，不断增加优质花生、优质大豆、饲料玉米和蓝莓、葡萄、张良姜、迷迭香、食用菌等优质农产品。大力发展现代生态循环农业，强化农业资源综合化利用，持续推进百亩千头生态方、绿色种植、生态养殖等发展计划。加快推动农村一二三产业融合发展，积极发展都市生态农业、休闲观光农业和“互联网+农业”，抓好健康食品产业园和韭菜科技、现代烟草科技等田园综合体建设；加强与泰国正大、大北农等企业合作，支持国润牧业、瑞亚牧业、康龙养殖等企业做大做强，推进农产品精深加工，培育壮大生猪、肉牛、大豆等八大农业产业化联合体。加快市农科院现代农业研发基地建设，不断提升农业科研支撑能力。切实做好非洲猪瘟等疫情防控工作，坚决防止重大疫情在我市发生。

深化农村综合改革。继续推进农村承包地“三权分置”改革，探索农村宅基地改革。稳步推进农村集体产权制度改革，完成农村集体资产清产核资，发展新型集体经济。持续深化、有序推进农村土地征收制度改革和农村集体经营性建设用地入市改革，加快农村产权交易市场和土地经营权抵押贷款试点建设。深化农村金融改革，强化农业保险保障作用。持续推进水利、供销社、农场、林权等改革，激发农业农村发展活力。

加快美丽宜居乡村建设。学习浙江经验，深入实施农村人居环境整治，抓好“千村整治、百村示范”，加快推进农村生活垃圾整治、生活污水处理、厕所革命、废弃物资源化利用、村容村貌提升等重点工作，新改建农村公路364公里，建设饮水安全巩固提升工程155处，解决和改善19万农村居民饮水安全问题，完成12万户农户改厕。加强农村诚信建设，保护好农村传统文化、乡土文化，鼓励村志编修和村史馆建设，推动农村移风易俗和精神文明创建。强化农村基层组织建设，深化拓展“四议两公开”工作法，创新乡村治理机制，提高乡村治理效能。开展全域国土综合整治，深入推进“大棚房”与农地非农化问题治理。

（七）强化基础能力建设，厚植经济发展优势。抢抓国家基础设施领域补短板政策机遇，加快实施一批重大项目，夯实发展基础，蓄积发展后劲。

加快综合交通体系建设。大力推进郑万铁路、三洋铁路、沙河复航、大西环、大南环、北环路升级及西延、平叶和平鲁快速通道、周南高速舞钢段、郑西高速鲁山段等项目建设，做好鲁山机场、许南公路东移、绕城高速以及焦唐、焦平高速平顶山段等项目前期工作。

加强水利基础设施建设。聚焦水资源、水生态、水环境、水灾害“四水同治”，加快编制全域循环生态水系规划，全力推进昭平台水库加固扩容、南水北调配套及连通、北部山体生态修复引水等十大工程，构建系统完善、丰枯调剂、循环畅通、多源互补、安全高效、清水绿岸的现代水利基础设施网络。

推进能源基础设施建设。深入实施电网补强工程，继续加大城乡配电网投资，加快推进50项重点电网项目建设，提升电网保障能力。加快发展清洁电源，持续抓好风电等可再生能源发电装机建设。新增供热面积130万平方米，新建天然气管网35公里，进一步提高供热、供气保障能力。

统筹生态系统建设。深入开展山水林田湖草一体化生态保护和修复，统筹建设森林、湿地、流域、农田、城市五大生态系统。按照“三增四转五统六化”要求，加快实施国土绿化提速行动，抓好廊道绿化、山区生态林、森林抚育、国家储备林建设等八项重点工程，完成营造林37.2万亩。深入开展河湖采砂专项整治行动，全面开展巡山巡矿行动，抓好北部山体、河湖采砂和工矿废弃地生态修复等重点工作，提升生态环境承载力。

强化信息网络建设。加强信息基础设施建设，推动资源整合和开放共享，推进我市5G建设和商用，加快大数据中心云资源池应用推广，开展中小企业和制造业上云专项行动，打通市、县、乡政务服务基础数据传输网络，努力实现数据互联互通。

（八）加强保障和改善民生，增进人民群众福祉。坚持以人民为中心，加大民生投入，办好民生实事，让人民群众有更多获得感、幸福感和安全感。

稳定扩大就业。把稳就业放在更加突出位置，加强就业统筹和岗位余缺调剂，实施失业保险援企稳岗“护航行动”。落实就业创业扶持政策，积极推进公共就业服务专项活动，持续实施全民技能振兴工程，促进高校毕业生、退役军人等重点群体多渠道就业创业，稳定农村劳动力转移就业，支持外出务工人员返乡下乡创业，加大对残疾人等就业困难人员援助力度，妥善做好“僵尸企业”处置和过剩产能化解中职工安置工作。

健全社会保障体系。大力实施全民参保计划，继续提高城乡居民基础养老金最低标准，继续调增企业和机关事业单位退休人员基本养老金。完善城乡居民医保门诊统筹制度，提高基本医保、大病保险、困难群众大病补充医疗保险保障水平。提高城乡低保、特困供养、孤儿养育等保障标准。加强残疾人权益保障，推进残疾人康复机构建设，按时足额发放残疾人两项补贴。严格落实抚恤优待政策，推进县级社会福利中心规范化建设。加强流浪乞讨人员救助。做好农村留守儿童关爱保护和困境儿童保护工作。加强社区规范化建设，提高社区服务水平。完善住房市场体系和住房保障体系，积极推进棚户区改造等保障性安居工程建设，推动房地产市场平稳健康发展。

优先发展教育事业。深入实施第三期学前教育行动计划，促进学前教育普及普惠发展，新建、改扩建幼儿园24所。编制义务教育阶段学校建设规划，完成消除大班额项目学校30所。持续建设农村寄宿制和小规模学校。规范校外培训机构。扎实推进高中阶段教育普及攻坚计划，改善普通高中办学条件。深入推进产教融合、校企合作和实训基地建设，支持高等院校转型发展。做好民办教育分类管理。加强教师队伍建设，改善教师工作生活条件，提高贫困地区乡、村、教学点教师生活补助标准，让教师成为最受尊敬的职业。加强综合治理，维护校园安全。健全学生资助制度，使更多孩子享受到公平而有质量的教育。

推进健康鹰城建设。健全分级诊疗和药品供应保障制度，推动基本医保支付方式改革。深化县域综合医改，建立紧密型县域医共体。加强区域全民健康信息平台建设，加快发展“互联网+医疗健康”，促进优质资源互联共享。做实家庭医生签约服务，完成乡村医生全员培训。大力支持社会办医。加强生育全过程健康服务，提高妇幼保健水平。抓好市第一人民医院门急诊楼、市第二人民医院病房综合楼、市精神卫生中心、市中心血站迁建等项目建设，筹建市妇幼保健院分院和区域医疗中心，不断优化医疗卫生服务。广泛开展全民健身活动，办好市第十届运动会暨第五届全民健身大会。落实“四个最严”要求，强化全过程监管，切实抓好食品药品安全，让人民群众吃得更放心、用药更安全。

促进文化发展繁荣。积极践行社会主义核心价值观，大力弘扬开放、包容、务实、创新的城市精神，抓好新时代文明实践中心建设，深化群众性精神文明创建。深度挖掘观音、曲艺、陶瓷、魔术、温泉等文化内涵，促进文化旅游融合发展，加快推进尧山文化旅游综合体、国家动漫产业发展基地平顶山园区、中国汝瓷博物馆、宝丰马街民俗园等项目建设，不断彰显文化凝聚力、增强文化竞争力。繁荣艺术创作，丰富文化生活。加强文物保护利用，做好非物质文化遗产项目申报，传承城市历史文脉。深入开展“扫黄打非”。推动市广播电视台机制改革，打造新型主流媒体，推进媒体融合发展。提高网络治理能力，打造清朗网络空间。

加强社会治理创新。学习借鉴“枫桥经验”，全面加强基层社会治理。开展阳光信访、责任信访、法治信访，促进人民群众合理合法利益诉求及时就地解决。深入开展普法教育，推进法制宣传教育进校园、进农村、进社区、进机关、进企业、进家庭。加强社会治安防控体系建设，高质量推进“一村（格）一警”工作，依法打击各种违法犯罪活动，推动扫黑除恶专项斗争纵深发展。扎实做好庆祝新中国成立70周年安保等工作，维护社会大局和谐稳定。

加强国防教育、国防动员和国防后备力量建设，支持国防和军队改革，支持驻平解放军、武警部队、预备役部队、民兵和人民防空建设。深入开展双拥共建，加强退役军人服务保障体系建设，争创全国双拥模范城“六连冠”。全面贯彻党的民族政策和宗教工作基本方针，加强民族宗教工作基层基础建设，促进民族团结、宗教和睦。支持工会、共青团、妇联等人民团体更好发挥桥梁纽带作用。做好外事、侨务、对台工作。开展好第四次全国经济普查和第三次全国国土调查。加强史志、档案、气象、地震、邮政等工作。

三、全面加强政府自身建设

面对发展新形势、人民新期待，政府工作要有新气象、新作为，必须更加注重加强自身建设，提升治理能力，加快建设人民满意的法治政府、创新政府、廉洁政府和服务型政府。

（一）坚持忠诚为政，把牢正确政治方向。高举习近平新时代中国特色社会主义思想伟大旗帜，坚决贯彻党的基本理论、基本路线、基本方略，树牢“四个意识”，坚定“四个自信”，坚决维护习近平总书记党中央的核心、全党的核心地位，坚决维护党中央权威和集中统一领导，真正把“两个维护”内化于心、外化于行，始终在政治立场、政治方向、政治原则、政治道路上同以习近平同志为核心的党中央保持高度一致。严格遵守政治纪律和政治规矩，坚持把党对一切工作的领导贯彻到政府工作各领域全过程，听从党中央号令，紧跟党中央步伐，政令畅通、令行禁止，不讲条件、不搞变通，确保习近平总书记重要指示批示和党中央大政方针贯彻落实不走样、不变形，决不允许不敬畏、不在乎、喊口号、装样子。

（二）坚持依法行政，加快建设法治政府。坚持依宪施政、依法行政，不断提高运用法治思维和法治方式深化改革、推动发展、解决问题、维护稳定的能力。坚持依法决策，健全完善程序，充分发扬民主，正确实行集中，对重大民生政策出台、重大公共项目建设要依法公示听证，进行深入咨询，组织专家论证，进一步提高决策科学、民主、法治化水平。深入推进政务公开，及时回应社会关切，确保权力在阳光下运行。依法接受人大法律监督和工作监督，自觉接受政协民主监督，认真办理人大代表建议和政协委员提案。强化审计监督，支持监察监督和司法监督，主动接受群众、社会和舆论监督。把诚信施政作为重要准则，确保部署落实到位、承诺兑现到位，切实增强政府公信力和执行力。

（三）坚持实干勤政，恪尽职守担当作为。弘扬焦裕禄同志的“三股劲”，加强学习研究，注重实践锻炼，在推进重点工作中积累经验，在解决重大矛盾中经受考验，在完成重要任务中增长才干，全面增强“八种本领”，有效解决不会为问题，着力增强善作为能力。始终保持锐意进取、永不懈怠的精神状态和敢闯敢干、一往无前的奋斗姿态，不回避矛盾，不惧怕挑战，不推卸责任，勇于挑最重的担子、啃最硬的骨头，敢于迎难而上，勤勉重干，真抓实干。健全容错纠错机制，激励干部担当作为，鼓励创造性贯彻落实。关心关爱基层一线干部，为担当的干部担当，对负责的干部负责，充分激发勇挑重担、苦干实干的活力动力。

（四）坚持从严治政，着力加强廉政建设。认真落实政府系统全面从严治党主体责任，严格落实中央八项规定及实施细则精神，深入开展电视问政活动，持之以恒纠治“四风”，反对空泛表态、应景造势、敷衍塞责和出工不出力，坚决纠正脱离实际的官僚主义、空喊口号的形式主义和简单粗暴的命令主义。持续加强党风廉政建设和反腐败工作，加强重点领域制度建设，强化审计和巡视巡察结果运用，盯紧公共资金、国资国企、政府采购、资源开发利用、公共资源交易等领域廉政风险点，扎牢织密制度笼子，铲除滋生腐败土壤，坚决查处贪腐行为。严格遵守廉洁自律准则，做到敬畏人民、敬畏组织、敬畏权力、敬畏法纪，筑牢防线、划清边线、不碰红线、守住底线，永葆为民务实清廉本色。

各位代表！

实干托起梦想，拼搏铸就辉煌。让我们更加紧密地团结在以习近平同志为核心的党中央周围，在市委坚强领导下，同心同德、砥砺奋进，加快推动平顶山综合实力高质量重返全省第一方阵，为中原更加出彩贡献力量、增添重彩，以优异成绩迎接中华人民共和国成立70周年！
